# A Boolean network of the crosstalk between IGF and Wnt signaling in aging satellite cells

**DOI:** 10.1371/journal.pone.0195126

**Published:** 2018-03-29

**Authors:** Lea Siegle, Julian D. Schwab, Silke D. Kühlwein, Ludwig Lausser, Stefan Tümpel, Astrid S. Pfister, Michael Kühl, Hans A. Kestler

**Affiliations:** 1 Institute of Medical Systems Biology, Ulm University, Ulm, Germany; 2 International Graduate School of Molecular Medicine, Ulm University, Ulm, Germany; 3 Leibniz-Institute on Aging – Fritz Lipmann Institute, Jena, Germany; 4 Institute of Biochemistry and Molecular Biology, Ulm University, Ulm, Germany; Duke University School of Medicine, UNITED STATES

## Abstract

Aging is a complex biological process, which determines the life span of an organism. Insulin-like growth factor (IGF) and Wnt signaling pathways govern the process of aging. Both pathways share common downstream targets that allow competitive crosstalk between these branches. Of note, a shift from IGF to Wnt signaling has been observed during aging of satellite cells. Biological regulatory networks necessary to recreate aging have not yet been discovered. Here, we established a mathematical *in silico* model that robustly recapitulates the crosstalk between IGF and Wnt signaling. Strikingly, it predicts critical nodes following a shift from IGF to Wnt signaling. These findings indicate that this shift might cause age-related diseases.

## Introduction

Aging is a highly complex biological process, which impacts health-related quality of life and life expectancy. The underlying mechanisms of aging are still poorly understood. Several theories have been postulated concerning the cause of aging. On a cellular level aging is, for instance, provoked by DNA damage, protein aggregation or cellular dysdifferentiation [1–5]. As a consequence, aging is commonly accompanied by a plethora of aging-related diseases such as cancer, neurodegenerative diseases, diabetes, osteoporosis and cardiovascular diseases [6]. Thus, a better understanding of the underlying pathways regulating life span serves as a basis to establish age-related therapy concepts.

IGF and Wnt signaling pathways have been linked to aging [7–10] and both signaling cascades share common downstream effectors. Wnt genes encode a highly conserved family of extracellular ligands. In vertebrates Wnt ligands form a family of 19 secreted glycoproteins which act either directly on the secreting cell (autocrine signaling) or indirectly on surrounding cells (paracrine signaling) [11–13].

Wnt molecules bind to seven-pass transmembrane Frizzled receptors and different co-receptors. Depending on the involved Wnt ligand, receptors and co-receptors, Wnt pathways are sub-divided into the canonical Wnt/β-catenin signaling cascade and the two non-canonical signaling branches Wnt/c-Jun N-terminal kinase (JNK) and Wnt/calcium pathway. Each Wnt pathway leads to distinct cellular responses. However, in satellite cells it could be shown that some Wnt ligands can regulate parts of canonical as well as non-canonical Wnt signaling [14,15]. Canonical Wnt/ β-catenin signaling induces β-catenin dependent transcription of pro-proliferative and pro-survival target genes. Non-canonical Wnt/JNK signaling modulates Ras-related C3 botolinum toxin substrate (Rac)- and small GTPase Rho (Rho)-mediated cytoskeletal rearrangements, thereby determining cell polarity and motility [11,16–20]. In contrast, non-canonical Wnt/calcium signaling activates calcium-dependent enzymes, thereby influencing gene expression, histone modification and cellular senescence together with their downstream targets [11,12,20,21].

Wnt signaling was shown to be deregulated during aging in diverse cell populations. For instance, in intestinal and hematopoetic stem cells a downregulation of canonical β-catenin signaling has been observed in aging [22,23]. In contrast, in a mouse-model of accelerated aging it was demonstrated that a loss of the Wnt antagonist Klotho increases Wnt signaling and triggers premature aging [8]. Moreover, satellite cell aging is accelerated by increased Wnt signaling whereas Wnt inhibitors revert the aged phenotype [5].

IGF signaling regulates growth, differentiation, survival and the metabolism of carbohydrates, proteins and lipids [10]. IGF initiates the PI3K-Akt-mammalian Target of Rapamycin (mTOR) pathway as well as the Rat sarcoma (Ras)-rapidly accelerated fibrosarcoma (Raf)-Mitogen-activated protein kinase (MAPK) signaling cascade. Protein Kinase B (Akt) and its downstream substrates inhibit pro-apoptotic molecules such as cyclin-dependent kinase inhibitors and Bad, thereby promoting cell survival [24–29]. In line with their pro-survival and pro-proliferative role in the Ras-Raf-extracellular signal-related kinase (ERK) cascade they are commonly up-regulated in several types of cancer [27,30,31]. As mTOR complexes 1 and 2 (mTORC1, mTORC2) influence cellular metabolism, a misregulation is associated with metabolic disorders such as diabetes and obesity [24,29,32–34].

There is extensive crosstalk between both IGF and Wnt signaling. The canonical Wnt signaling mediator β-catenin not only interacts with TCF/LEF transcription factors but also with FoxO, which is regulated by IGF signaling [7,11,12,16,17,21,25,35–37]. The important negative regulator of Wnt signaling, axin 2, has also been shown to be a target gene of IGF signaling [38]. On the other hand, components of IGF induced Ras-Raf-MAPK signaling can be induced by Dishevelled (Dvl), a component of Wnt signaling. This suggests a complex cross regulatory network between IGF and Wnt signaling [39–41]. Interestingly, in satellite cells and muscle cells Wnt signaling increases with aging [5], while IGF signaling decreases [42] and the crosstalk of both pathways influences aging and aging related diseases [43,44].

This raises the questions whether the known interactions between both pathways can be represented in a mathematical model and whether this model subsequently can represent changes during aging of satellite cells. Such a model of the dynamics and cross regulation of IGF and Wnt signaling might be beneficial for a better understanding of the mechanisms underlying aging.

Boolean networks are a powerful tool to model dynamic cellular signaling pathways. They make use of the assumption that a gene is either expressed or not, resulting in two states: ON and OFF [45–51].

All activating and repressing influences on a molecule are abstracted and summarized in its Boolean function. This Boolean function is then used to determine the subsequent state of all molecules of the model. To simulate a Boolean model usually an initial state is either given or randomly generated. Starting from this initial state, the states of the molecules are updated via the Boolean function creating a sequence of states until the model enters a circle of recurring states. Recurring states are called attractors and may represent known stable states. They are linked to biological phenotypes. Attractors may either consist of a series of states or just one single recurring state [45–47].

The aim of this work was to create a Boolean network model, which accurately describes the crosstalk between IGF and Wnt signaling as it was observed in satellite cells. The knowledge incorporated into this model originates from extensive literature research. The model was then used to simulate the interaction of both IGF and Wnt signaling in the context of aging.

## Materials and methods

### Boolean networks

Boolean networks were introduced as dynamic mathematical models to simulate gene regulatory processes by Stuart Kauffman in 1969 [52]. In Boolean networks only two states are discriminated for each regulatory factor—active (1/TRUE/ON) or inactive (0/FALSE/OFF). Additionally, a Boolean transition function (using operators like AND/OR/NOT) is specified for each regulatory factor. These functions describe the dynamics of the network. Whether a regulatory factor is active or not can be determined by applying the corresponding activation function. The state of the Boolean network at a specific time point *t* is specified by a binary string containing the value of each regulatory factor at that specific time point. In synchronous Boolean networks the state of each regulatory factor is updated at the same time. Consequently, a state transition is performed by synchronously applying all Boolean functions. A Boolean network with *n* regulatory factors has 2^*n*^ possible states—one state for each possible combination of gene values. The state space can be depicted as a directed graph with one node for each state in the network. In this graph called state graph the transition from one state to another is represented by directed edges between the nodes.

The state space of a Boolean network is finite. Consequently, consecutive state transitions irrespective of the initial state eventually converge to a single state or a cycle of states called attractor. Attractors describe the long-term behavior of a Boolean network and can be associated with biological phenotypes [53]. Each of the states has to be examined to exhaustively search for all attractors in the state space. Albeit their simplicity, Boolean networks have proven to be valid models for regulatory processes such as human oncogenic pathways [49], embryonic cardiac development [48], mammalian cell cycle [54] and cholesterol regulatory signaling [55].

Biological systems are considered to be robust against perturbations [56]. Computer intensive tests were performed to examine whether the attractors or Boolean functions in the created model were significantly more stable than random networks.

### Modeling and simulation setup

The Boolean network model for the IGF/Wnt crosstalk was derived from collecting literature data. Modeling of the network functions was performed using ViSiBooL [57]. The exhaustive attractor search simulation to analyze the network was carried out with the R-package BoolNet [58].

To further analyze the effects of IGF/Wnt crosstalk in our network model, we divided the model in its IGF and Wnt sub-networks. Therefore, we removed the crosstalk elements from the Boolean functions. The attractors of the sub-networks were associated to those of the crosstalk model. This was done by comparing the overlapping regulatory factors of the complete network and the IGF and Wnt sub-network respectively in all the attractors. Two attractors are matched, if all overlapping factors are equal. The IGF/Wnt crosstalk model consists of 23 nodes. Therefore, there are 2^23^ = 8,388,608 possible start states.

## Results

### A Boolean model of the IGF/Wnt crosstalk

**Fig 1:**
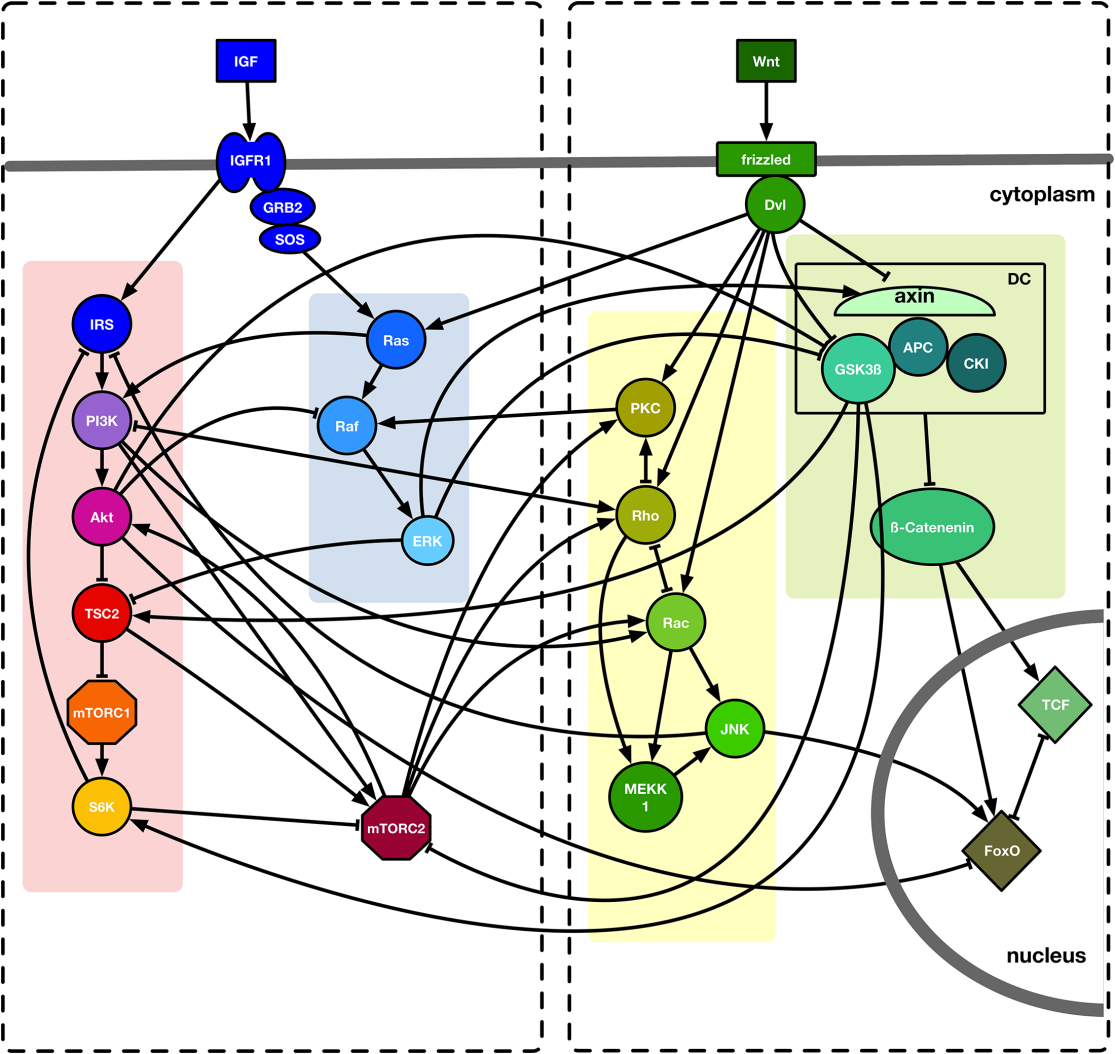
Crosstalk of IGF and Wnt signaling. IGF and Wnt signaling are simplified and reduced to their most important nodes. Signaling pathways are highlighted in different colors and the IGF and Wnt sub-networks are depicted by the dashed boxes. Interactions between two molecules are symbolized as black lines. Activation is represented by arrowheads, inhibition by bar-headed arrows. Cellular compartments are separated by grey bars.

To construct a regulatory network of the crosstalk between IGF and Wnt, we collected published data of both pathways and incorporated the core molecules into our model. An overview of this model and its internal interactions is given in Fig 1.

Cellular signaling pathways are tightly controlled and depend on external stimuli. IGF and Wnt molecules can affect cells in a paracrine manner. Not necessarily being secreted by the Wnt or IGF affected cells themselves, they cannot be activated within the network. Therefore, in our model IGF and Wnt are considered inputs. Here, we briefly describe the rules implemented to model IGF and Wnt signaling and list the simplifications made within our approach.

Canonical Wnt signaling is activated by Wnt binding to a Frizzled receptor (Fzd) and the co-receptor Lipoprotein receptor-related protein 5/6 (LRP5/6). This receptor complex in turn activates Dishevelled (Dvl) [11,12,59]. In the non-canonical Wnt/JNK pathway, Dvl is activated in a similar manner, although a different co-receptor, the Receptor tyrosine kinase-like orphan receptor (Ror), is used [17]. However, to simplify the model both Fzd/LRP5/6 and Dvl are omitted and downstream targets of them are activated by Wnt itself. Wnts can also activate the small GTPases Rac and Rho [12,16–18,60,61]. Rac is responsible for cell polarization and the formation of lamellipodia, whereas Rho leads to cell contraction due to the phosphorylation of actomyosin and light-chain myosin [62]. Due to this contrary function of Rac and Rho on the cytoskeleton, they cannot be active at the same time [19,63–65]. Both Rho and Rac can further activate Mitogen activated protein kinase kinase kinase 1 (MEKK1) [30,66]. To activate Wnt/calcium signaling a Wnt ligand activates a G-protein coupled to a Frizzled receptor and Dvl in order to stimulate Phospholipase C (PLC), the latter of which hydrolyses Phosphatidylinositol-(4,5)-bisphosphate into diacylglycerol (DAG) and Inositiol-(1,4,5)-trisphosphate [12,17]. This facilitates calcium influx and in turn DAG can further activate Protein kinase C (PKC) [25]. This step is simplified in our model in a manner that Wnt molecules can activate PKC. Additionally, PKC can be activated downstream of Rho [24,32,67–69]. PKC is able to inhibit Rho, thereby creating a negative feedback loop [70,71].

β-catenin, the key effector of canonical Wnt signaling, can be phosphorylated and primed for proteasomal degradation by a destruction complex (DC) including the glykogen synthase kinase 3 beta (GSK3β) and axin 2, which triggers β-catenin inactivation [11,12,59]. Dvl (here via Wnt) inhibits GSK3β and consequently β-catenin is stabilized and can translocate into the nucleus where it activates the transcription factors of the T-cell factor family (TCF) or Forkhead Box-O (FoxO) [7,11,12,16,17,21,25,35–37].

Binding of IGF to its receptor IGFR1 results in its activation and creates a Src homology 2 (SH2) binding site for activators of Ras [10,27,30,72,73]. In the next step Ras, which can also be activated by Dvl (here via Wnt) [39–41], activates Raf [27,30,73–76] and this then activates ERK [30,31,73,77,78]. To facilitate the model, IGF can directly activate nodes downstream of IGFR1.

In addition, activated IGFR1 (here via IGF) can also activate intracellular targets like Insulin receptor substrate 1 (IRS) [10,71]. Phosphoinositide 3 kinase (PI3K) binds to IRS and recruits Phosphoinositide-dependent kinase (PDK) [24,68,79,80]. The first link of IGF to a regulation of lifespan was identified with the *Caenorhabditis elegans* gene *age-1*, being homologous to PI3K [9,81]. PDK is required for the activation of Akt [24,68,79]. To simplify our model, we assumed that IRS activates PI3K and this further activates Akt. Another activator of PI3K is Ras [26,63,82–84]. PI3K influences the cytoskeleton by activating Rho and Rac [84–89]. A downstream target of Rho termed Rho-associated protein kinase (ROCK) activates phosphatase and tensin homolog (PTEN), which is an inhibitor of PI3K. As a consequence, a negative feedback loop is created [20,24,26,27,80,82,90]. Here, we included this step by assuming that Rho inhibits PI3K.

Downstream of PI3K Akt inhibits Tuberous Sclerosis Factor 2 (TSC2) by phosphorylation [24,80,91,92], thereby activating mTORC1, which is otherwise inhibited by TSC2 [24,68,79,91,92]. ERK can inhibit TSC2 as well [24,93]. The activated mTORC1 further activates p70-S6 kinase (S6K). To avoid excessive IGF signaling a feedback inhibition of IRS by S6K is initiated [24,27,32,69,79,91]. Besides, S6K is an inhibitor of mTORC2 [24,27,32].

The conditions for mTORC2 activation are not yet completely understood. Association of the complex with ribosomes through PI3K seems to be necessary [28,32,68,86,94] as well as active TSC2 [68,79,86,92,95]. GSK3β is able to phosphorylate and thereby activate TSC2. This is dependent on an adenosine monophosphate-activated protein lowering mTORC2 activity when cellular energy is sparse [59,68,91–93,96]. This node is included in our model by assuming that GSK3β inhibits mTORC2.

In addition, GSK3β activates S6K [24,33,68,79,80,91,97,98]. GSK3β can also be inhibited by ERK or Akt [27,40,80,99–101]. Likewise, Raf can be inhibited by Akt. Another cross regulation is the activation of axin 2 by ERK [38]. Besides the activation through PI3K, Akt can be activated by mTORC2 through phosphorylation [24,29,68,79,91]. Moreover, mTORC2 activates PKC and thus is able to manipulate the cytoskeleton via Rho and Rac [68,69,88,95,98].

A very common interaction of the Wnt/calcium pathway and IGF signaling is the activation of Raf by PKC [24,30,66,76,100,102,103] while Raf activation through canonical Wnt signaling is assumed [40,72]. As part of non-canonical Wnt signaling Rac is able to activate JNK [12,17,20,60,85,102]. Another way of activating JNK is mediated by MEKK1 [30,31,77,78]. By inhibiting IRS JNK is able to reduce IGF signaling [24,33,96]. Both FoxO and JNK are part of the cellular stress response. Hence, it is not surprising that JNK is a strong activator of FoxO [7,25,35,99,104,105]. TCF requires β-catenin to function as a transcription activator [106]. However, FoxO is also able to bind β-catenin and act as a transcription factor [35]. Thus, they have to compete for binding of β-catenin and their activation is mutually exclusive. Consequently, they inhibit each other [103,104].

JNK is a potent activator of FoxO and thus contributes to the inhibition of TCF via FoxO. FoxO is a transcription factor for Rictor, which is a component of mTORC2. Since FoxO-dependent transcription of Rictor only boosts mTORC2 activity but cannot activate mTORC2 itself we did not include FoxO in the regulation of mTORC2 and further activation of Akt. However, Akt can phosphorylate FoxO and thus inhibit its function as a transcription factor. Thereby, it is acting as a negative feedback regulator [25,26,29,80,99,107]. In the network, we only included the inhibition of FoxO by Akt.

The Boolean functions corresponding to the previously described regulatory interactions are depicted in Table 1.

**Table 1:** Table of the Boolean functions of the IGF/Wnt crosstalk model.

Node	Boolean function
Wnt	Wnt
axin	ERK | !Wnt
GSK3β	!(Wnt | ERK | Akt)
DC	axin & GSK3β
β-catenin	!DC
TCF	β-catenin & !(JNK & FoxO)
FoxO	!Akt & β-catenin
Rho	(Wnt | PI3K | mTORC2) & !(Rac | PKC)
Rac	(Wnt | PI3K | mTORC2) & !Rho
MEKK1	Rac | Rho
JNK	MEKK1 | Rac
PKC	Rho | Wnt | mTORC2
IGF	IGF
IRS	IGF & !(S6K & JNK)
PI3K	(IRS | Ras) & !Rho
Akt	PI3K | mTORC2
TSC2	!(Akt | ERK) | GSK3β
mTORC1	!TSC2
S6K	mTORC1 | GSK3β
Ras	IGF | Wnt
Raf	(Ras | PKC) & !Akt
ERK	Raf
mTORC2	!(S6K | GSK3β) & (PI3K | TSC2)

Abbreviations used: DC, destruction complex; GSK3β, Glycogen synthase kinase 3 beta; TCF, T-cell specific transcription factor; FoxO, Forkhead Box-O; Rho, small GTPase Rho; Rac, Ras-releated C3 botulinum toxin substrate; MEKK1, Mitogen activated protein kinase kinase kinase 1; JNK, c-Jun N-terminal kinase; PKC, Protein kinase C; IGF, insulin-like growth factor; IRS, Insulin receptor substrate 1; PI3K, Phospahtidyl-inositide 3 kinase; Akt, Protein kinase B; TSC2, Tuberous Sclerosis Complex 2; mTORC1, mammalian target of rapamycin complex 1; S6K, p70-S6 kinase; Ras, Rat sarcoma; Raf, rapidly accelerated fibrosarcoma; ERK, the Ras-Raf-extracellular signal-related kinase; mTORC2, mammalian target of rapamycin complex 2; &, and; |, or; !, not

### Attractors of the IGF/Wnt crosstalk model

**Fig 2:**
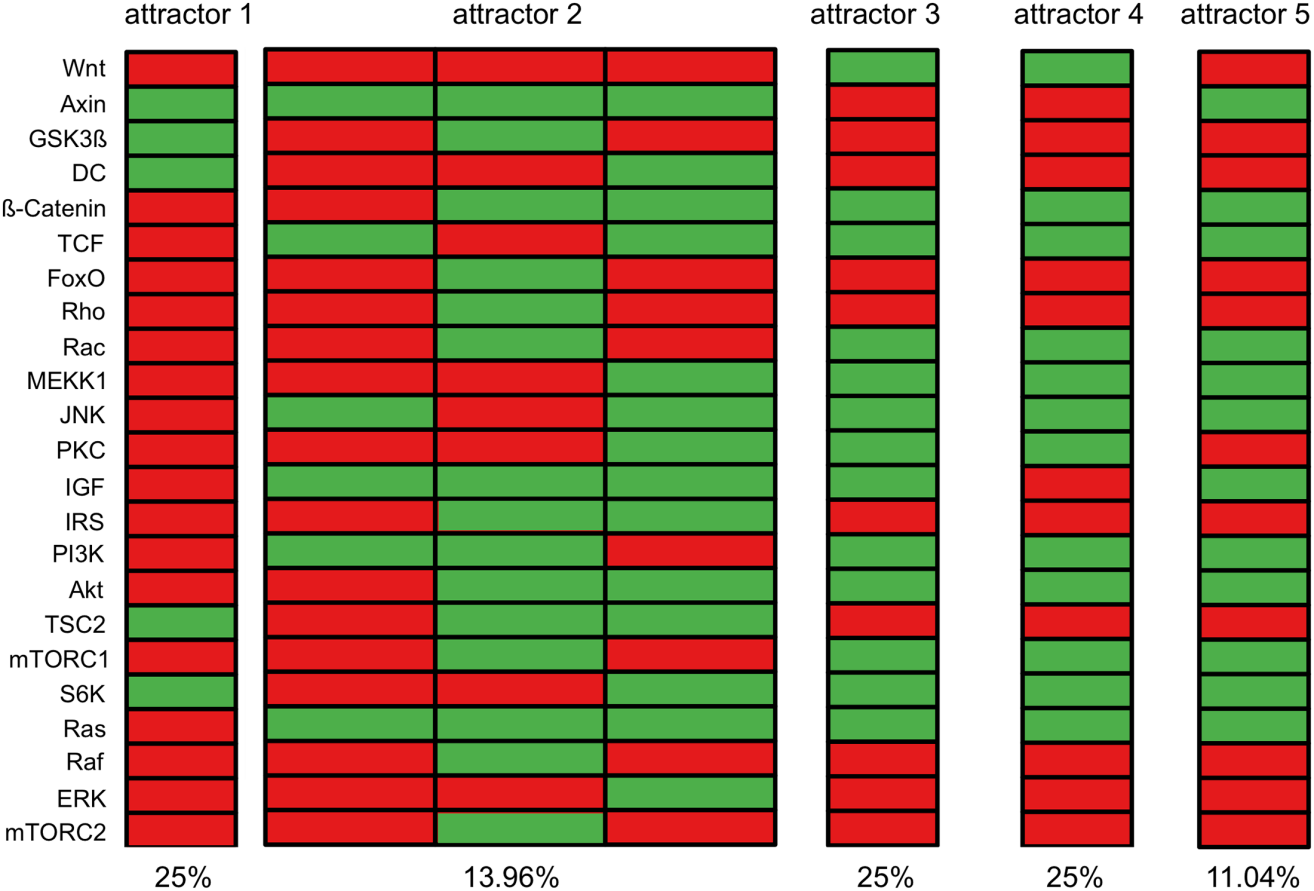
Attractors of the IGF/Wnt crosstalk model. Exhaustive attractor search of the IGF/Wnt crosstalk model yielded four single state attractors and one three-states attractor. The frequency of occurrence of each attractor is given as percentage below each column. Each block represents an attractor. The nodes are listed on the y-axis. Each rectangle symbolizes the state of a node: red stands for inactive, green for active.

Once the model of the IGF/Wnt crosstalk was established by the transformation of literature statements into Boolean functions we performed an exhaustive search to identify all attractors of the model. Five attractors named attractor 1 to attractor 5 were identified with different frequency (Fig 2). Four of these attractors were single-state attractors and one was a cyclic attractor with three states.

Depending on the initial state the nodes representing the Wnt and IGF inputs stay active or inactive over the entire time period. Thus, there are four possible combinations of external input factors for our model: I) both IGF and Wnt are active (attractor 3), II) only IGF or only Wnt is active (attractors 2 and 5, respectively attractor 4), or III) both are inactive (attractor 1).

Attractor 1 represents the state of an un-stimulated cell; neither IGF nor Wnt are active and thus no input is given. During aging IGF signaling slowly declines while both canonical and non-canonical Wnt signaling increases [43]. Based on this we assumed a stimulation by IGF only results in a young phenotype (attractors 2 and 5) and stimulation by Wnt an aged phenotype (attractor 4).

However, when starting a simulation from an initial state where all nodes except the input factors are off, every attractor could be reached, except for attractor 5. Since attractor 5 could not be reached starting from the chosen initial state it was excluded from further analysis. Attractors 3 and 4 represent the age-related shift from IGF to Wnt signaling. Due to the slow transformation process first both IGF and Wnt are active (attractor 3) and afterwards only Wnt as external input is active (attractor 4).

### Signaling cascade of an un-stimulated cell

Simulations of a biological network by computational Boolean models should resemble the *in vivo* situation as closely as possible. Therefore, the created model is supposed to follow the same temporal expression pattern as during biological IGF/Wnt crosstalk.

**Fig 3:**
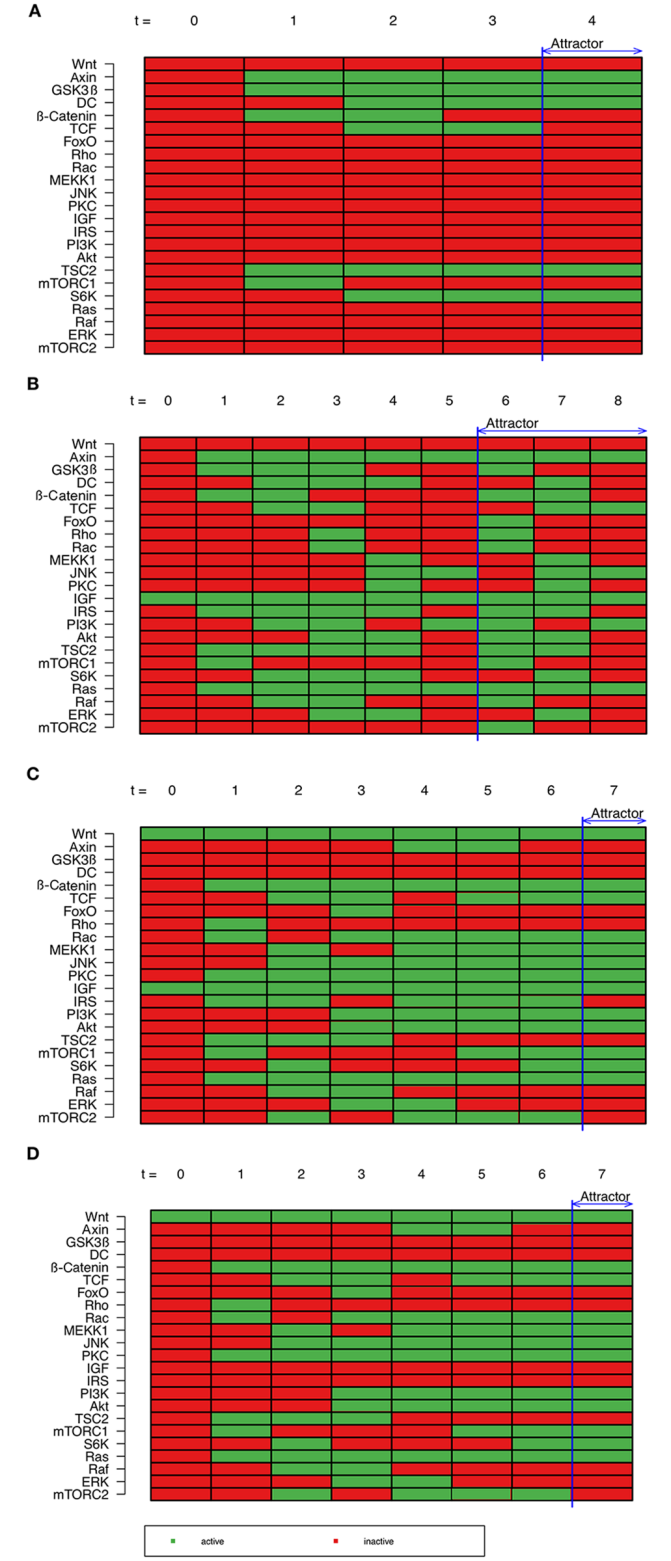
Effects of input factors in signaling cascade. (A) Based on an initial state where all nodes are inactive, a simulation of a signaling cascade was performed. The model results in an attractor representing an un-stimulated cell. (B) Simulation from an initial state with IGF as single active node results in an attractor representing the young phenotype. (C) In contrast, a simulation of signaling cascade with IGF and Wnt as single active nodes results in an attractor representing a mid-aged phenotype. (D) Simulation of the signaling cascade with Wnt as single active node results in an attractor representing an aged phenotype. Nodes are listed on the y-axis. Time is plotted on the x-axis. Every rectangle represents the state of a node at a specific time: red stands for inactive, green for active.

IGF and Wnt are extracellular signaling molecules and thus cannot be activated from within the network. If the simulation of the signaling cascade starts with an initial state where all nodes are inactive (Fig 3A), the network reaches a single-state attractor (attractor 1). This attractor represents an un-stimulated cell. Here, GSK3β is active because it is ubiquitously expressed and stays active if not otherwise inhibited. Downstream targets of GSK3β are S6K and TSC2 which both can be activated by GSK3β resulting in an active state in this attractor.

### Signaling cascade of IGF—A young phenotype

Starting from an initial state in which all nodes are inactive expect the external input IGF, the simulation of a signaling cascade reaches the three-states attractor 2 (Fig 3B). Here, IGF initiates the Ras-Raf-MAPK-cascade leading to ERK activation. Though PI3K-Akt cascade is initially inactivated due to a negative feedback, it is partially active in the attractor. Influence of the crosstalk on Wnt signaling is among others the activation β-catenin and TCF as well as Rho, Rac, PKC and JNK.

As this attractor consists of three states, it probably does not represent any fixed state of a cell but rather the transition from one cellular state to another. Here, IGF and Ras as well as the canonical Wnt antagonist axin 2 are the only nodes that are active in all three states. All other nodes switch between their active and inactive state during iterations. This represents crosstalk and internal regulations of the signaling pathways too avoid permanent activation of signaling cascades. For instance it was previously demonstrated that IRS is inhibited by S6K to avoid an excess of IGF signaling [24,27,32,69,79,91].

As mentioned before, IGF signaling is more active at the beginning of a lifespan and declines with time [43]. Therefore, this attractor represents a young phenotype.

### Signaling cascade of the of IGF and Wnt—A mid-aged phenotype

Simulation of a signaling cascade starting from an initial state in which only the two external input factors IGF and Wnt are active (Fig 3C), the model reaches a single state attractor. In this signaling cascade the canonical Wnt signal disrupts the destruction complex and β-catenin is activated. However, its downstream target TCF is not active in the entire signaling cascade. TCF competes with FoxO for the binding to β-catenin. Due to the activation of Akt by IGF, FoxO gets inactivated and thus TCF is again active in the attractor. During aging, IGF signaling declines whereas Wnt signaling increases [43]. However, this shift proceeds slowly and therefore the resulting attractor from the signaling cascade of IGF in combination with non-canonical Wnt signaling represents a mid-aged phenotype.

### Signaling cascade of Wnt—An aged-phenotype

Originating from a cell with active Wnt as single active node (Fig 3D), canonical Wnt signaling disrupts the destruction complex and β-catenin is activated. Despite the inactive IGF, TCF is not active over the entire signaling cascade. Here, it competes again with FoxO until it is inactivated by Akt due to crosstalk.

In addition, Ras-Raf-MAPK signaling is also activated by crosstalk, even if IGF is not present. This crosstalk is mediated by factors of non-canonical Wnt signaling such as PKC, Rac and JNK.

The resulting attractor is a single state attractor with active canonical and non-canonical Wnt signaling as well as components of IGF signaling. Wnt signaling increases with age [43]. Therefore, this attractor represents an aged phenotype.

### Modeling the age-related shift from IGF to Wnt signaling

During aging of muscle cells, IGF signaling slowly declines while Wnt signaling increases [43]. Also, non-canonical Wnt signaling shifts from non-canonical to canonical signaling [14,108,109]. For this reason, a shift from IGF to Wnt signaling was simulated. In a non-aged satellite cell IGF signaling is active. Therefore, a simulation based on an initial state in which all nodes are switched off expect IGF as external input was performed (Fig 3B). After five time periods the signaling cascade reaches an attractor. This attractor is the circulating three-state attractor (attractor 2) with IGF as single extracellular input. With respect to aging, this circulating state is maintained until an unknown signal leads to a shift from IGF to Wnt signaling.

**Fig 4:**
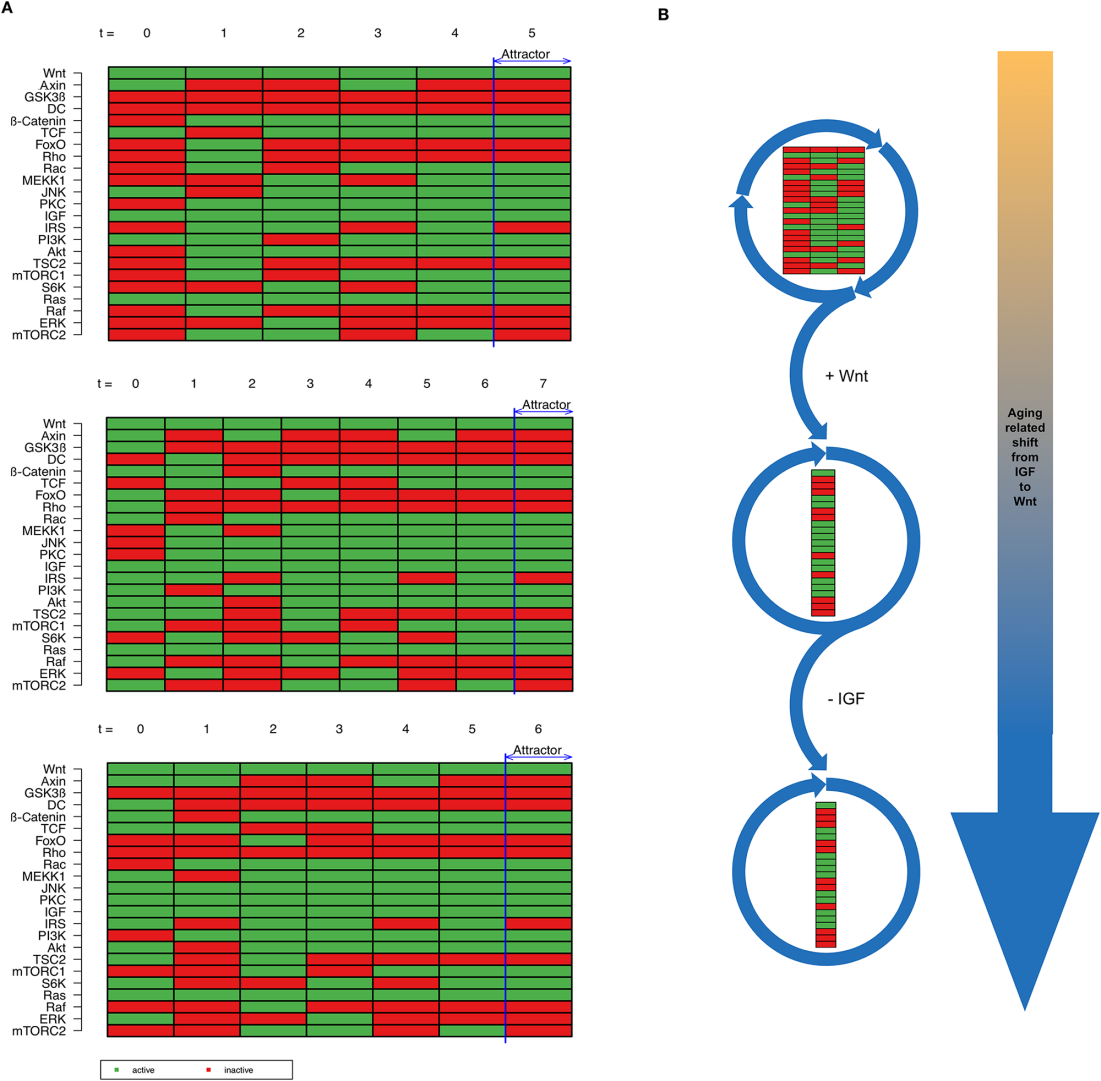
Age-related shift from IGF to Wnt signaling. (A) The age-related shift from IGF to Wnt happens stepwise. At the beginning of this shift both signals are active and the temporal sequence simulation results in a single state attractor. (B) Passing the life span of an organism, initially IGF as external signal is active, resulting in a three-state attractor. Then, a slow shift from IGF to Wnt takes place. At the beginning both input factors are active, whereas at the end Wnt as single external input is active, resulting in a single-state attractor.

Afterwards, the shift from IGF to Wnt signaling was simulated by adding Wnt as an additional input factor. However, a slow shift from IGF to Wnt is supposed to occur. Therefore, we first activated Wnt as external input of the model in addition to IGF and performed another simulation based on the states of the prior attractor 2. This time the model reached attractor 3 (Fig 4A). It turned out that it does not matter from which initial state out of three possible states from attractor 2 we started our simulation. Each simulation resulted in attractor 3 (Fig 4A). As a result of this simulation we could show that switching from IGF to Wnt signaling changes the behavior of the model.

In a last step we turned off IGF signaling and simulated a signaling cascade with only Wnt as external input, thereby reaching attractor 4 (Fig 4B). The effect of aging results in different attractors, which is explained in the following section.

#### Imbalance between IGF and Wnt signaling in favor of Wnt

During aging of muscle cells, IGF signaling slowly declines while Wnt signaling increases [43] and thus IGF and Wnt are simultaneously active before Wnt signaling outweighs IGF signaling. Attractor 3 represents this transformation from IGF to Wnt signaling. Here, both external input factors IGF and Wnt are present. However, attractor 3 closely resembles attractor 4 with Wnt as the only extracellular input signal. The only difference between these two attractors is the activity of IGF. IGF signaling is self-limited by a negative feedback-loop from S6K to IRS [110,111]. This can be seen in attractor 2 and in the signaling cascade in Fig 3B. In combination with Wnt IRS is doubly inhibited by S6K and additionally JNK [112,113]. However, in the signaling cascades of Wnt we could already show that downstream targets of Wnt are also able to induce downstream components of the IGF signaling by crosstalk such as PI3K and Akt. Consequently, there is an imbalance between IGF and Wnt signaling in favor of Wnt.

Of note, the aged phenotype might be prone to develop aging-related diseases. Here, TCF is currently active and can further induce the expression of the canonical Wnt target gene cyclin D [114,115]. Thereby it promotes cell cycle progression and enables cancer formation if deregulated. Furthermore, Akt is permanently active in this attractor. It deactivates pro-apoptotic proteins like Caspase-9 or Bad as well as cell cycle inhibitors p21 and p27. Akt additionally inhibits GSK3β [116,117] and thus further promotes the activation of TCF. All these events drive cancer formation, progression and invasiveness. Moreover, S6K is active in both attractors 2 and 3. S6K can be activated by mTORC1, which is also active in these attractors. IRS can be inhibited by S6K resulting in insulin-resistance thereby promoting the development of diabetes mellitus type II [118,119]. S6K also stimulates autophagy which is increased in type II diabetes [120].

### The aging process can only be represented through the IGF/Wnt crosstalk

**Fig 5:**
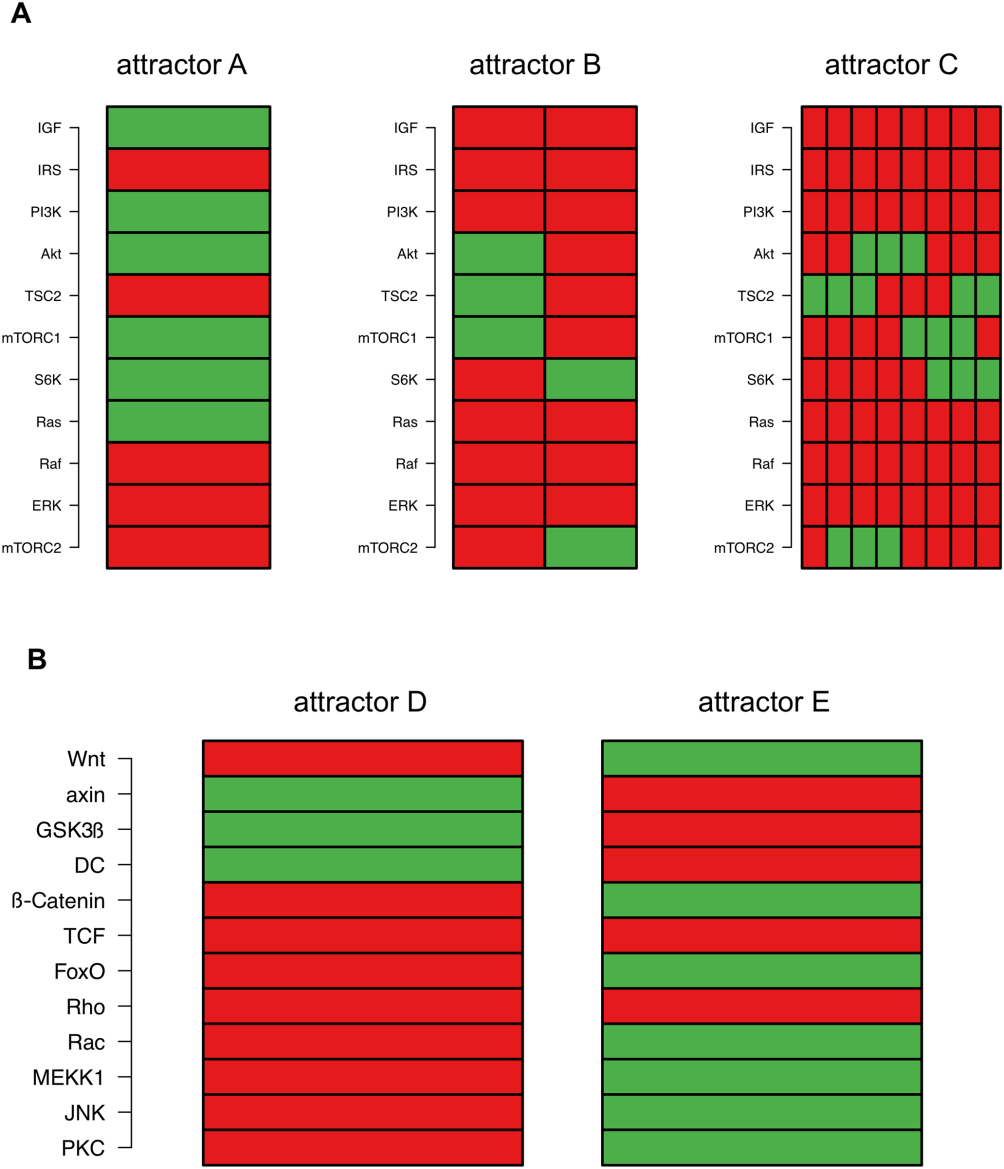
Attractors of the sub-networks. (A) Simulation of the IGF sub-network lead to attractors A, B and C, the first of which could be matched to attractors 3 and 5 of the complete crosstalk model (see Fig 2). (B) Attractors D and E were found while simulating the Wnt sub-network. Here, attractor D could be matched to attractor 1. Each block represents an attractor. The regulatory factors are listed on the y-axis. Each rectangle symbolizes the state of such a factor: red stands for inactive, green for active.

To investigate the relevance of the IGF/Wnt crosstalk in our model we have simulated both pathways separately. As seen in Fig 1 (dashed boxes), we divided the whole network into IGF components and Wnt components without their crosstalk interactions (transition functions can be found in S1 Text) and performed an exhaustive attractor search (Fig 5).

The simulation of the IGF sub-network leads to three attractors (Fig 5A), called attractors A, B and C. Matching these three attractors to the crosstalk network shows that the pattern of attractor A can be found in both attractors 3 and 5 (Fig 2). On the other hand, the crosstalk present in the complete model seems to stabilize the network and thus the patterns of attractors B and C no longer exist. Two attractors called attractor D and E (Fig 5B) were found when simulating the Wnt sub-network. Of these two, attractor D could be matched to attractor 1 of the crosstalk model (Fig 2). As with the IGF sub-network the crosstalk seems to stabilize the network leading to a removal of pattern of attractor E. However, the sub-network simulations did not reveal attractors 2 and 4, which were found in the crosstalk model, representing young and old phenotypes with respect to aging.

This leads to the conclusion that the modeling of the aging process of satellite cells with the IGF/Wnt crosstalk model is only possible through the interaction of both pathways while the simulation of only one of the two pathways could not realize aging.

### Robustness analysis of IGF/Wnt crosstalk model

Biomolecular networks are considered to be robust. This means that a perturbation of single molecules in most cases does not influence the behavior of the system. We evaluated the robustness of both the IGF/Wnt crosstalk model as well as the sub-networks.

Biological networks can adapt to environmental changes and their functions are resistant to damage [121]. The network models’ robustness was evaluated in terms of their transition robustness. The transition robustness of the models is determined using a computer-intensive test. The test perturbs states in the network with a random bit flip, which corresponds to a point mutation in the biological context. In a second step, the corresponding successor state of the original state as well as of the perturbed state is computed. Then, the distance between the two successor states is measured using the normalized Hamming distance. The Hamming distance computes the number of genes which differ between the original successor state and the successor state of the perturbed network. To compare networks of different sizes the Hamming distance is then normalized by the number of genes in the network. The distance shows how well a Boolean network can maintain its functionality under mutation conditions. A normalized Hamming distance of zero indicates that the mutation has no effect on evaluated network behavior. This test was repeatedly done for 100 randomly drawn states of the Boolean network model and the mean normalized Hamming distance was computed, denoted by H. Additionally, this was tested with 1000 randomly generated networks. A comparison to the results of the random networks indicated if the constructed Boolean network model is significantly more robust than a randomly generated network of the same size.

**Fig 6:**
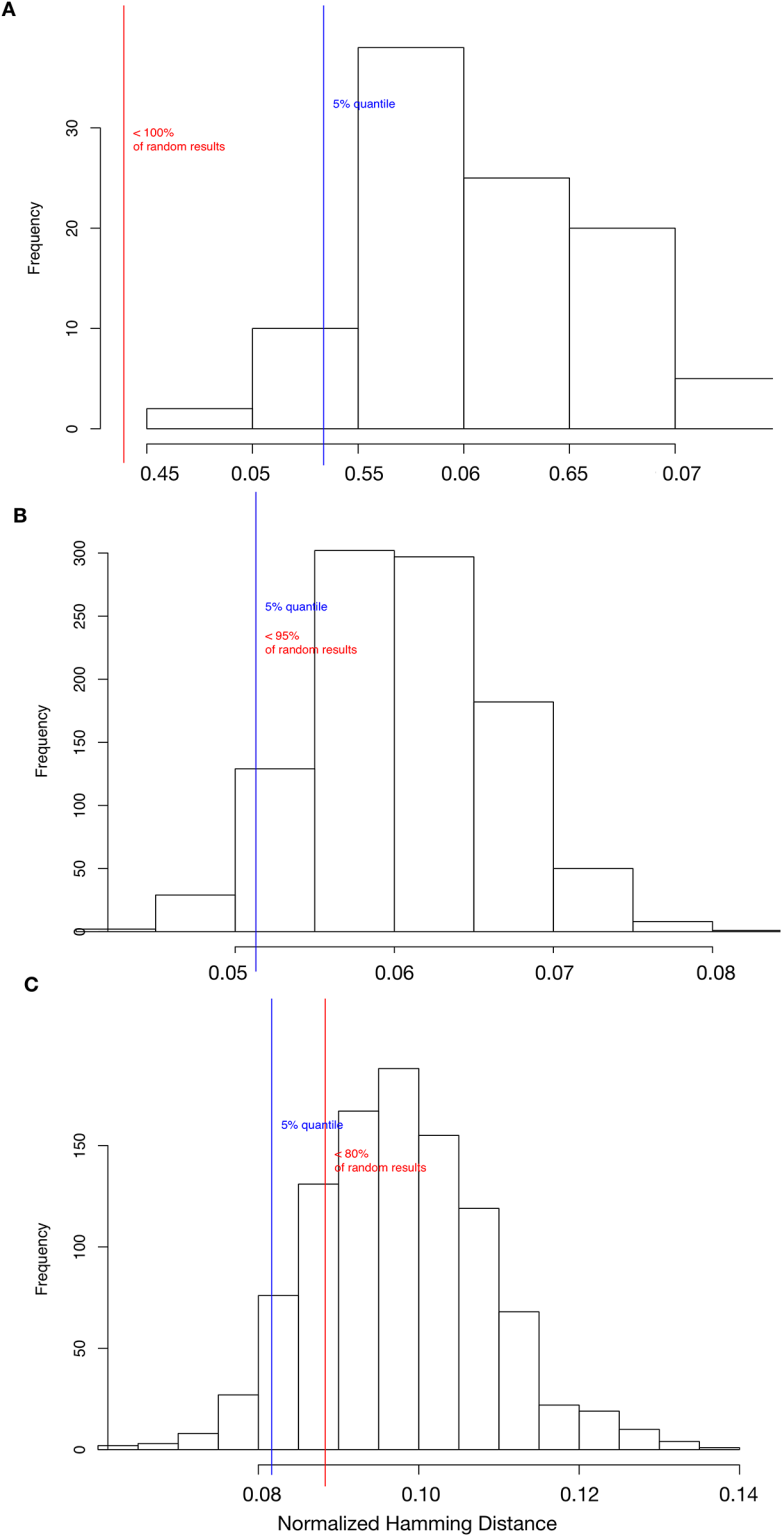
Transition robustness. (A) 100 randomly drawn states of the IGF/Wnt model were mutated by bit flip (point mutation) and their successor states were computed. The successor states of the mutated and the original states were then compared using the normalized Hamming distance (red line). The same was done for 100 randomly generated networks of the same size (histogram). The blue line shows the 95% quantile. (B) shows the same test for the IGF sub-network and (C) for the Wnt sub-network.

In addition to the crosstalk model, this computer-intensive test was performed for each of the two sub-network models. The results were then compared (Fig 6). The IGF/Wnt crosstalk model shows a statistically significant result of H = 0.043 (p < 10^6^) in comparison to the randomly generated networks (Fig 6A). A mean normalized Hamming distance of 0.043 can be interpreted as 4.3% of the genes in the states are differing in the mutated networks on average. The IGF sub-network shows a mean normalized Hamming distance of H = 0.05 (p = 0.051, Fig 6B). Fig 6C shows a mean normalized Hamming distance of H = 0.088 (p = 0.2) in the Wnt sub-network, which is not significantly smaller than in the randomly generated networks.

## Discussion

In this study, we created a model of the molecular interactions between IGF and Wnt signaling in satellite cells and muscle aging by integrating more than 80 publications of the IGF/Wnt research field. It was demonstrated that the model recreates the behavior of the signaling pathways and viable cellular conditions in form of temporal sequences and attractors. In addition, the *in silico* model can predict and recapitulate *in vitro* and *in vivo* experiments.

The simulated signaling pathways of IGF and Wnt behave as described in literature. However, knowledge concerning the interaction of IGF and Wnt signaling as a whole is still missing. Mostly, there is a description of single molecule interactions and their impact on another signaling pathway. But often these interactions are not yet fully understood. Thus, the importance of individual interactions for the behavior of a network is hard to judge. Our model demonstrates that there is a crosstalk between IGF and Wnt signaling. This can be seen by the activation of non-canonical Wnt signaling by IGF in attractor 2 or by the activation of PI3K-Akt and Ras by Wnt signaling in attractor 4.

A circulating state with dynamic regulation between the single nodes of the network is only achieved with IGF as single input. Once Wnt signaling is introduced into the model (attractor 3 and attractor 4) the network stabilize and the simulation ends in a single state attractor which favors the behavior of Wnt signaling. As mentioned before, IGF signaling shifts to Wnt signaling during aging of satellite cells. By use of the model we demonstrate that the imbalance between these two pathways potentially leads to aging-related diseases. In particular, the permanent activation of TCF and Akt can lead to the development of cancer due to cell cycle activation and inhibition of apoptosis. Another well-known age-related disease is diabetes mellitus type II. It is defined by the reduced response of insulin receptors regarding normal insulin concentrations. A common underlying mechanism for the development of diabetes is the dysregulation of IRS [119]. In our model IRS is only active in two time periods in attractor 2 representing the young and healthy state whereas it is switched off in attractor 3 and attractor 4 which represent an aged phenotype. Thus, the potential of developing diabetes mellitus type II is probably related to the shift of IGF to Wnt signaling during aging.

The simulation of IGF and Wnt pathways separately did not yield the young and old phenotype attractors (attractor 2 and attractor 4), which could be found in the IGF/Wnt crosstalk model. This further supports our thesis that crosstalk between IGF and Wnt pathways exists. Furthermore, the crosstalk is necessary to represent the aging process whereas Wnt or IGF signaling on their own are not sufficient to describe the aging process.

Beside IGF and Wnt, there is a multitude of other growth factors and external stimuli, which can also influence the behavior of a cell. It stands to reason that signaling molecules and pathways other than IGF and Wnt interact with each other and shape the behavior of these two signaling pathways. Especially the simulation of the IGF/Wnt shift supports this theory. As aging is a highly complex process, IGF and Wnt signaling might certainly not be the only players involved.

Despite being a commonly examined pathway, Wnt signaling is still not fully understood. Examples for this are the activation of the mTOR complexes or the activation of canonical and non-canonical Wnt signaling pathways by different Wnt molecules. Especially the latter is dependent on the cell type. Our model was simplified to include the main signaling pathways.

During aging, the functionality of stem cells decreases in adult tissues [22,122], which seems to be driven by alterations in stem cell self-renewal pathways [22]. Especially the canonical Wnt/β-catenin signaling pathway, one of these self-renewal pathways, is important for the maintenance of stem cells [17,123]. Alterations in the Wnt signaling pathway play a role in aging processes of adult tissues [5,8]. However, the alterations of Wnt signaling pathway differ between several stem cell populations. For satellite cells it was shown that an increased activity of canonical Wnt/β-catenin signaling pathway accompanied by a decrease in IGF signaling leads to dysfunctions during aging [5,42]. In contrast, a decline of canonical Wnt/β-catenin signaling was noticed in intestinal and hematopoietic stem cells during aging [23,124]. In intestinal stem cells a balance between Wnt and Notch signaling instead of IGF and Wnt signaling regulates differentiation and maintenance of stem cells [125], which both decline with age [124]. An increase in canonical Wnt signaling can even rejuvenate intestinal stem cells [124].

It can be concluded that there are different mechanisms in Wnt signaling in different cell types. There are 19 Wnt proteins encoded in the human genome [126], which can bind to one or more of the over 15 known Wnt receptors and co-receptors [127]. Depending on the Wnt protein and the receptor combination different downstream signaling cascades are activated and is not yet fully elucidated, which combination leads to which cascade. It is important to note, that thepresented model depicts the situation during aging of satellite cells, where the shift from IGF to Wnt takes place.

After constructing and simulating our model, we evaluated its dynamic behavior by confirming the results with published laboratory experiments which were not included in the model construction. We could indeed confirm that IGF activates Akt, S6K and ERK [128] which are downstream targets of IGF signaling cascades (Fig 1). This can be seen in attractor 2 (Fig 2). We can see in our model that IRS is mostly inactive due to negative feedback inhibition. In the mid-aged phenotype, IRS is inhibited by co-inhibition via S6K and JNK (as described above), while downstream targets of IGF signaling like PI3K/Akt are still active due to crosstalk activation. However, our results are consistent with the results from von Maltzahn et al. who found a cross-activation of IGF downstream targets (e.g. PI3K, Akt and S6K) by Wnt7a independently of IGF receptor activation [15]. Increased activation of Wnt and its downstream targets (e.g. β-catenin and TCF) in aging satellite cells—as simulated in our model—was also found in Naito et al. [44] and Watanabe et al. [129].

Biomolecular networks are usually robust, as the perturbation of single molecules does not change the behavior of the system, at least not drastically. To test the robustness of the IGF/Wnt crosstalk model we compared the model to 1000 randomly generated networks of the same size. The transition robustness measures the influence of point mutations on the network. An analysis of the transition robustness of the crosstalk model, reveals that the IGF/Wnt crosstalk model is significantly more robust than randomly generated networks of the same size (p < 10^6^). In contrast, transition robustness of the sub-network models of Wnt (p = 0.051) and IGF (p = 0.2) does only borderline or not differ statistically from random networks. Comparison of the transition robustness of the IGF/Wnt crosstalk model (mean 4.3% differences in states after mutation, Fig 6A) and the IGF (5%, Fig 6B) and Wnt sub-network models (8.8%, Fig 6C) also indicated that the crosstalk model is more robust. These results show that the crosstalk of the IGF and Wnt pathways increases the robustness against perturbations such as point mutations. Hence, we can conclude that the crosstalk between the IGF and Wnt signaling leads to a stabilization of the network.

This model of IGF and Wnt signaling pathways and their molecular interactions represents an initial model. As research progresses IGF and Wnt signaling will be better characterized and new players, regulatory connections and interactions will be discovered. This novel knowledge can easily be incorporated into this initial model to refine and expand it.

Taken together, this model of IGF and Wnt signaling is able to reproduce the basic behavior of IGF and Wnt signaling and displays the interactions between these two signaling pathways even during aging in satellite cells. The model can be used to gain insight into the connections of these signaling pathways. It is also able to uncover novel aspects where knowledge is missing to correctly simulate the behavior of IGF and Wnt signaling in cells. As such, this model functions as a predictor for future targets.

## Supporting information

S1 Text Boolean functions of IGF and Wnt subnetwork models without crosstalk. (PDF)
