# Supplementary material for: A Boolean network of the crosstalk between IGF and Wnt signaling in aging satellite cells
Source: PLoS One. 2018 Mar 29;13(3):e0195126. doi: 10.1371/journal.pone.0195126 (PMC5875862; doi:10.1371/journal.pone.0195126)
Supplement: S1 Text — (PDF) [file pone.0195126.s001.pdf]

S1 Boolean functions of IGF and Wnt subnetwork models without crosstalk

Dependencies of the IGF subnetwork without crosstalk elements

```
## Boolean network with 11 genes
##
## Involved genes:
## IGF IRS PI3K Akt TSC2 mTORC1 S6K Ras Raf ERK mTORC2
##
## Transition functions:
## IGF = IGF
## IRS = IGF & !S6K
## PI3K = (IRS | Ras )
## Akt = PI3K | mTORC2
## TSC2 = !(Akt | ERK)
## mTORC1 = !TSC2
## S6K = mTORC1
## Ras = IGF
## Raf = Ras & !Akt
## ERK = Raf
## mTORC2 = !(S6K) & (PI3K | TSC2)
```

Dependencies of the Wnt subnetwork without crosstalk elements

```
## Boolean network with 12 genes
##
## Involved genes:
## Wnt axin GSK3b DC b_catenin TCF FoxO Rho Rac MEKK1 JNK PKC
##
## Transition functions:
## Wnt = Wnt
## axin = !Wnt
## GSK3b = !(Wnt)
## DC = axin & GSK3b
## b_catenin = !DC
## TCF = b_catenin & !(JNK & FoxO)
## FoxO = JNK
## Rho = Wnt & !(Rac | PKC)
## Rac = Wnt & !Rho
## MEKK1 = Rac | Rho
## JNK = MEKK1 | Rac
## PKC = Rho | Wnt
```
